# Highly Multiplexed Single-Cell In Situ RNA and DNA Analysis by Consecutive Hybridization

**DOI:** 10.3390/molecules25214900

**Published:** 2020-10-23

**Authors:** Lu Xiao, Renjie Liao, Jia Guo

**Affiliations:** Biodesign Institute & School of Molecular Sciences, Arizona State University, Tempe, AZ 85287, USA; lxiao15@asu.edu (L.X.); Renjie.Liao@asu.edu (R.L.)

**Keywords:** fluorescence in situ hybridization, FISH, transcripts, genomic loci, transcriptomics, genomics

## Abstract

The ability to comprehensively profile nucleic acids in individual cells in their natural spatial contexts is essential to advance our understanding of biology and medicine. Here, we report a novel method for spatial transcriptomics and genomics analysis. In this method, every nucleic acid molecule is detected as a fluorescent spot at its natural cellular location throughout the cycles of consecutive fluorescence in situ hybridization (C-FISH). In each C-FISH cycle, fluorescent oligonucleotide probes hybridize to the probes applied in the previous cycle, and also introduce the binding sites for the next cycle probes. With reiterative cycles of hybridization, imaging and photobleaching, the identities of the varied nucleic acids are determined by their unique color sequences. To demonstrate the feasibility of this method, we show that transcripts or genomic loci in single cells can be unambiguously quantified with 2 fluorophores and 16 C-FISH cycles or with 3 fluorophores and 9 C-FISH cycles. Without any error correction, the error rates obtained using the raw data are close to zero. These results indicate that C-FISH potentially enables tens of thousands (2^16^ = 65,536 or 3^9^ = 19,683) of different transcripts or genomic loci to be precisely profiled in individual cells in situ.

Academic Editors: 

## 1. Introduction

Highly multiplexed single-cell in situ nucleic acids analysis promises to provide new insights into many fields in biology and medicine, such as neuroscience, cancer biology and precision medicine [1]. Next-generation sequencing [2,3] and microarray technologies [4] are powerful tools to profile nucleic acids on a transcriptome- or genome-wide scale. Nevertheless, as these approaches require the nucleic acids to be extracted from the cells and subsequently purified before sequence identification, the cellular location information of the transcripts and genomic loci is lost during analysis. Multicolor karyotyping technologies [5,6,7,8] have been developed to visualize chromosomes in their natural spatial contexts. However, these approaches have not been applied for profiling transcripts or genomic loci. Their multiplexing capacities are also limited. Single-molecule fluorescence in situ hybridization (FISH) [9] and templated fluorescence turn-on probes [10] allow individual transcripts or genomic loci to be visualized in single cells. Nonetheless, due to the broad absorption and emission peaks of the common fluorophores, their spectral overlap limits the number of nucleic acids that can be quantified simultaneously in the same specimen. 

To enable spatial transcriptomics and genomics analysis, a number of methodologies have been investigated, such as combinatorial labeling [11,12,13], reiterative hybridization [14,15,16,17], in situ sequencing [18,19], sequential hybridization [20,21,22], and multiplexed error-robust FISH (MER-FISH) [23,24,25]. Although these methods allow a large number of nucleic acids to be profiled in situ, some nonideal factors still exist. For instance, to permit transcriptome- or genome-wide analysis, the multiplexing capacity of combinatorial labeling and reiterative hybridization needs to be further improved. As a result of its low efficiency of reverse transcription reactions, in situ sequencing can only detect a small fraction of the transcripts. Consequently, RNA species with low copy numbers may not be detectable. In each cycle of SeqFISH and MER-FISH, not all transcripts are stained. As a result, an artificial threshold has to be determined for signal recognition. However, due to the imperfect hybridization efficiency and also the varied number of probe binding sites on transcripts from the same RNA species, any thresholds will lead to some false positive and false negative signals. These errors could be corrected by doing an increased number of hybridization cycles. However, more errors will be generated in these additional cycles, leading to the more complicated data analysis and correction process. 

We report here a novel spatial transcriptomics and genomics approach. This method applies consecutive fluorescence in situ hybridization (C-FISH) to stain each nucleic acid molecule. As a result, every transcript or genomic locus is detected as a fluorescent spot, which has a fixed cellular location but different colors in the varied hybridization cycles. This generated unique color sequence is used to decipher the identity of the transcript or genomic locus. The multiplexing capacity of our approach is determined by the number of the different color sequences, which increases exponentially with the number of C-FISH cycles. Therefore, with just a small number of hybridization cycles, our approach will enable comprehensive single-cell in situ RNA and DNA analysis. To demonstrate the feasibility of this method, we show that transcripts or genomic loci can be successfully detected with 2 dyes in 16 cycles or with 3 dyes in 9 cycles. The error rates generated from raw data is close to “0”. With the high multiplexing capacity and low error rate, our approach will enable tens of thousands (2^16^ = 65,536 or 3^9^ = 19,683) of varied RNA species or genomic loci to be accurately quantified in single cells in their natural spatial contexts.

## 2. Results

### 2.1. Platform Design

**Figure 1:**
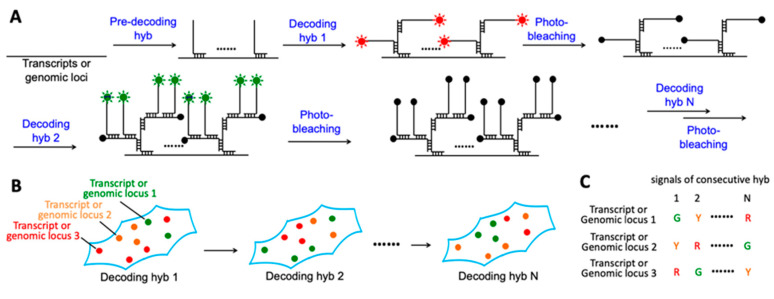
C-FISH: a novel method for spatial transcriptomics and genomics analysis. (**A**) Each C-FISH cycle is composed of three major steps, including probe hybridization, fluorescence imaging and photobleaching. The red and green sun-like symbols represent excited fluorophores, and the black solid dots represent photobleached fluorophores. (**B**) Under a microscope, every nucleic acid molecule is visualized as a fluorescent spot in each analysis cycle. (**C**) With their locations remaining throughout all the cycles, the identities of different nucleic acids can be determined by their corresponding color sequences.

In this C-FISH approach (Figure 1A), an individual nucleic acid target is first hybridized with a set of pre-decoding probes. These probes are not fluorescently labeled and have varied target-binding sequences. The pre-decoding probes hybridized to the same target also have the shared decoding sequence, which will recruit the fluorescently labeled decoding probes. Due to the reiterative cycles of washing or probe stripping in MER-FISH and SeqFISH, some pre-decoding probes could be dehybridized from their target, leading to a reduced signal to noise ratio as the cycle number increases. Consequently, the error rates will become more significant in the latter cycles. To address this issue, each decoding probe in our approach has one binding site to hybridize to the probe in the former cycle and two binding sites to recruit two probes in the next cycle. In this way, the staining intensities and the signal to noise ratio will increase with the cycling number. In each analysis cycle, after the hybridization of the decoding probes, each individual transcript or genomic locus is detected as a fluorescent spot under a fluorescence microscope (Figure 1B). Following image capture and data storage, all of the fluorescence signals are erased by photobleaching. As the nucleic acid targets are chemically crosslinked to other cellular components, their locations and the corresponding fluorescent spots will remain in place in every analysis cycle. With consecutive cycles of hybridization, imaging and photobleaching, the identities of each individual transcript or genomic locus is determined by their unique color sequences (Figure 1C). The number of different color sequences increases exponentially with the cycling number. For instance, with M fluoropohores used in every cycle and a total of N analysis cycles, an overall of M^N^ transcripts or genomic loci can be quantified in the same sample in situ. 

### 2.2. Efficiency and Effects of Photobleaching

**Figure 2:**
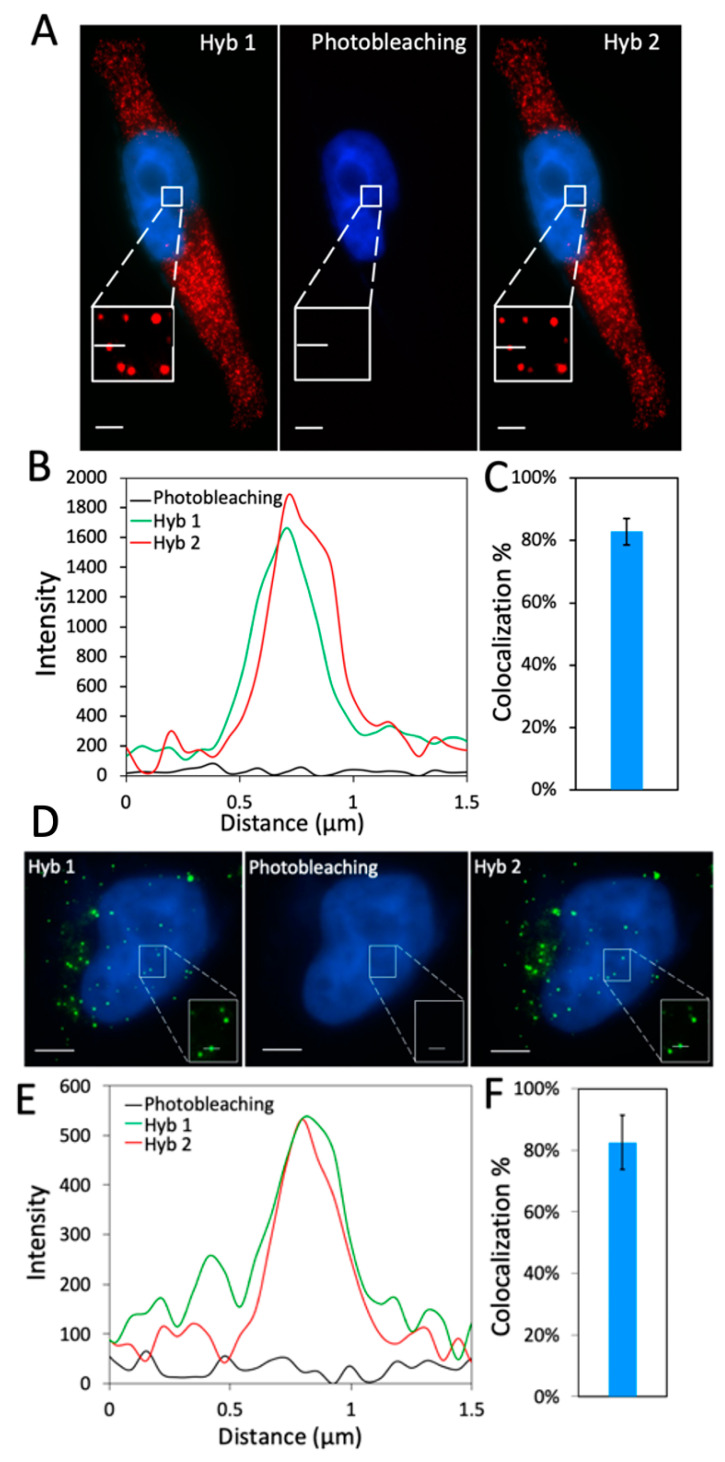
(**A**) GAPDH transcripts in HeLa cells are detected with Cy5 labeled decoding probes in two cycles of C-FISH. (**B**) Signal intensity profiles corresponding to the line positions in (A). (**C**) Fraction of GAPDH spots identified in the first cycle of hybridization that reappeared in the second cycle (*n* = 30 cells). (**D**) Ki67 transcripts in HeLa cells are detected with Quasar 570 labeled decoding probes in two cycles of C-FISH. (**E**) Signal intensity profiles corresponding to the line positions in (**D**). (**F**) Fraction of Ki67 spots identified in the first cycle of hybridization that reappeared in the second cycle (*n* = 30 cells). Scale bars, 5 µm.

For the success of this C-FISH approach, two critical requirements exist. First, the target staining signals generated in each analysis cycle must be removed with high efficiency. As a result, the leftover signals from the previous cycles will not lead to false positive results in the following cycles. In addition, the nucleic acids integrity should not be damaged during this signal removal process, and all the pre-decoding and decoding probes should remain hybridized to their specific targets. In this way, the RNA or DNA molecular targets can be restained and visualized in the same location in the subsequent cycles. Our group has demonstrated that photobleaching can effectively remove the fluorescence signals while maintaining the integrity of nucleic acids [14]. For these reasons, here we apply photobleaching to erase the fluorescence signals. To evaluate whether the probes remain hybridized to their targets after photobleaching, we stained mRNA GAPDH and Ki67 (Figure 2) with two cycles of C-FISH. After photobleaching, almost all the fluorescence signals are removed. Upon the hybridization of the second cycle decoding probes, the GAPDH and Ki67 transcripts are successfully restained (Figure 2A,B,D,E). We counted the stained transcripts in 30 cells and observed that over 80% of fluorescent spots identified in the first cycle reappear in the second cycle (Figure 2C,F). These results are consistent with the ones generated by staining the same transcript with 2 color FISH probes [5]. The small fraction of spots that do not reappear in the second cycle may be attributed to the non-specifically bound probes. To exclude these false positive signals, we consider only the spots colocalized in the first two hybridization cycles as real signals, which correspond to the target staining. This choice of real signals is also justified by the results in Section 2.3. The results here confirm that photobleaching can efficiently remove the fluorescence signals without damaging the nucleic acids integrity, and also indicate that all of the applied probes remain hybridized to their targets throughout the analysis cycles. 

### 2.3. Multi-Cycle C-FISH for RNA Analysis

**Figure 3:**
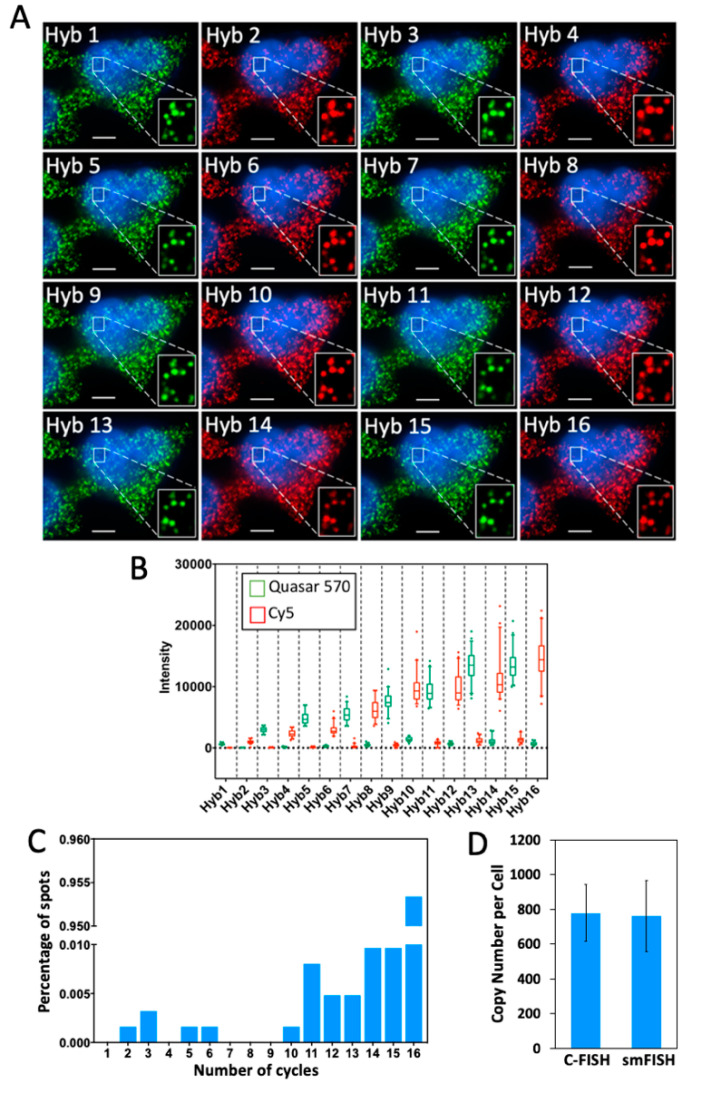
(**A**) GAPDH transcripts in HeLa cells are detected in 16 cycles of C-FISH. Quasar 570 and Cy5 labeled decoding probes were used in the odd and even hybridization cycles, respectively. (**B**) Intensity distribution of the GAPDH transcripts (*n* = 45 spots) in 16 cycles of C-FISH. In each cycle, the signal intensities in Quasar 570 and Cy5 channels are shown in green and red, respectively. (**C**) Fraction of GAPDH spots (*n* = 1000 spots) appear in different number of C-FISH cycles. (**D**) Comparison of the GAPDH mean copy number per cell (*n* = 30 cells) of transcripts measured by C-FISH and conventional single molecule FISH (smFISH) (*p* > 0.58). Scale bars, 5 µm.

**Figure 4:**
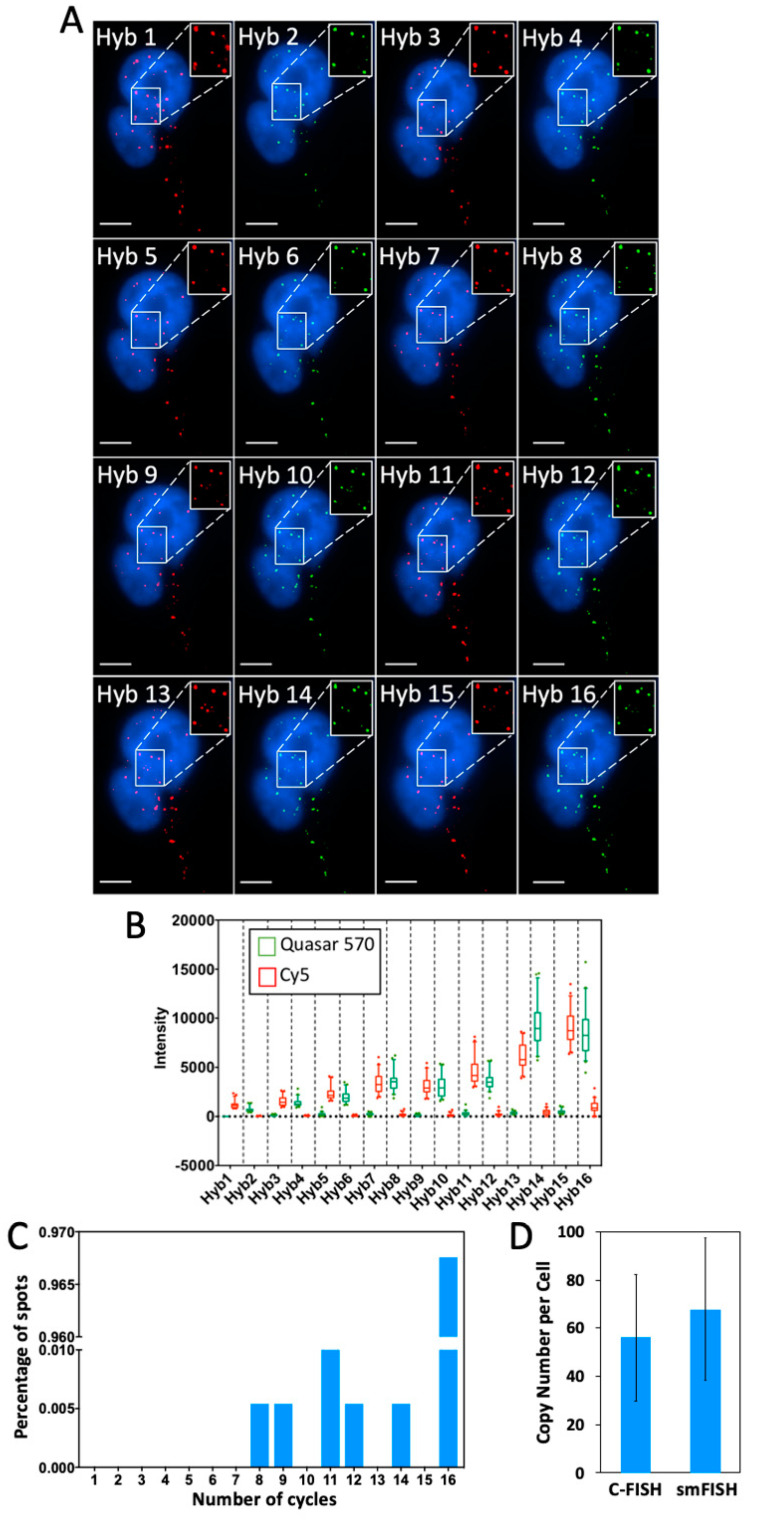
(**A**) Ki67 transcripts in HeLa cells are detected in 16 cycles of C-FISH. Cy5 and Quasar 570 labeled decoding probes were used in the odd and even hybridization cycles, respectively. (**B**) Intensity distribution of the Ki67 transcripts (*n* = 45 spots) in 16 cycles of C-FISH. In each cycle, the signal intensities in Quasar 570 and Cy5 channels are shown in green and red, respectively. (**C**) A fraction of Ki67 spots (*n* = 1000 spots) appear in different number of C-FISH cycles. (**D**) Comparison of the Ki67 mean copy number per cell (*n* = 30 cells) of transcripts measured by C-FISH and conventional single molecule FISH (smFISH) (*p* > 0.52). Scale bars, 5 µm.

To evaluate the multi-cycle potential of C-FISH for RNA analysis, we stained mRNA GAPDH (Figure 3) and Ki67 (Figure 4) with Quasar 570 and Cy5 in 16 consecutive hybridization cycles. The fluorophores on the decoding probes were switched in every cycle, to facilitate the evaluation of the signal to background ratio. We quantified the fluorescence intensities of the FISH spots in both the Quasar 570 and Cy5 channels in each cycle (Figure 3B and Figure 4B). As the cycle number increases, the staining intensities are enhanced, while the background remains largely the same. This increased signal to background ratio is attributed to the fact that every single probe applied in the previous cycle can recruit multiple probes in the next cycle. Additionally, due to such design, over 99.8% of the spots colocalized in the first two cycles reappear in the third cycle, and more than 95% of those colocalized spots are successfully detected in all of the following 14 cycles (Figure 3C and Figure 4C). These results further justify our choice of only using the spots colocalized in the first two cycles as real signals. In comparison, with all the probes degraded by DNase in SeqFISH, only ~78% of spots colocalized in the first two cycles are visualized in the third cycle [16]. We calculated the transcript copy number per cell using the number of spots detected in all of the 16 cycles, and compared our results with the ones generated by conventional single molecule FISH (smFISH) (Figure 3D and Figure 4D). The results provided by these two methods and those reported using RNA-seq [22] closely resemble each other. These results suggest that C-FISH allows accurate analysis of mRNA in single cells in situ. 

### 2.4. Multi-Cycle C-FISH for DNA Analysis

**Figure 5:**
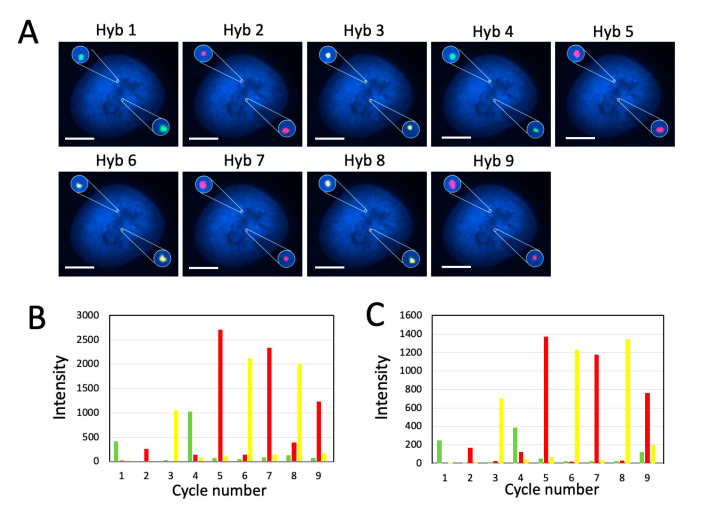
(**A**) Genomic locus 4p16.1 in HeLa cells (*n* = 30 cells) are detected in 9 cycles of C-FISH. Alexa 488 labeled decoding probes were used in the 1st and 4th cycles. Cy5 labeled decoding probes were used in the 2nd, 5th, 7th and 9th cycles. Quasar 570 labeled decoding probes were used in the 3rd, 6th and 8th cycles. (**B**) Signal intensity profiles of the upper spot in (A). (**C**) Signal intensity profiles of the lower spot in (A). The signal intensities in Alexa 488, Quasar 570, and Cy5 channels are shown in green, yellow and red, respectively. Scale bars, 5 µm.

To assess whether C-FISH can be applied for DNA analysis, we stained genomic locus 4p16.1 with Alexa 488, Quasar 570 and Cy5 in nine hybridization cycles (Figure 5). As thermal denaturation has destructive effects on chromatin [26], C-FISH only performs such treatment once to denature the double-stranded DNA targets at the beginning of the assay, which is required for all of the FISH-based DNA analysis methods. In the following cycles, decoding probes are hybridized directly to the probes in the previous cycle, eliminating the requirements of the repeated thermal denaturation processes. In each analysis cycle, the genomic locus was unambiguously detected in all the analyzed cells (*n* = 30 cells) (Figure 5A). We quantified the signal intensities of the two FISH spots shown in Figure 8A in all the three fluorescence channels. Throughout the nine cycles, only the signals from the correct fluorescence channels were observed; the fluorescence intensities in the other two channels were just around the background level (Figure 5B,C). In the first several cycles, the staining intensities are amplified, as an increasing number of decoding probes are recruited to stain the target. In the latter cycles, the signal intensities are slightly decreased. The reason for that might be that the bulky environment in the nucleus limits the number of the hybridized decoding probes. The results here indicate that C-FISH enables DNA molecules to be successfully quantified by a color barcode. And with the large number of the varied color sequences in the barcodes, highly multiplexed analysis of genomic loci can be achieved in single cells in situ. 

### 2.5. Multi-Cycle C-FISH for Multiplexed RNA Analysis

**Figure 6:**
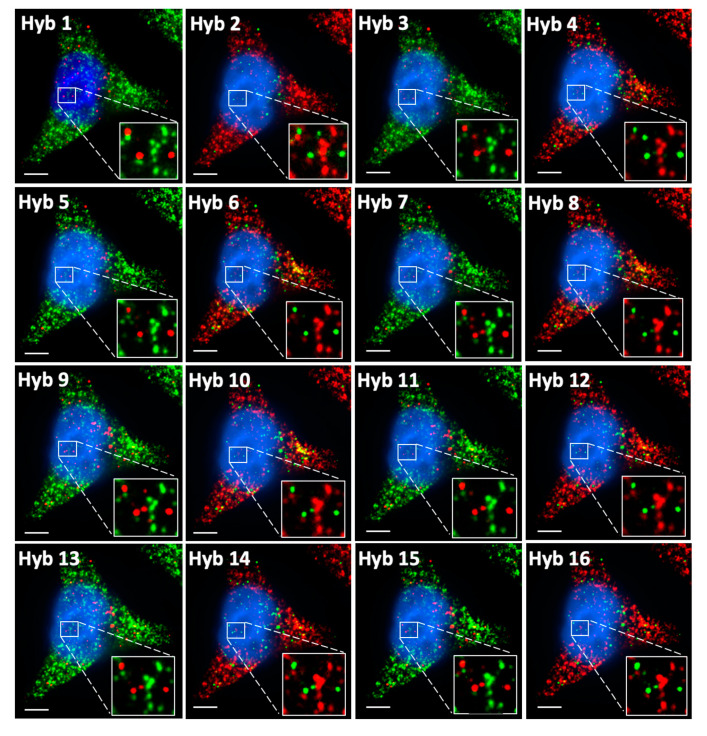
GAPDH and Ki67 transcripts in HeLa cells are detected simultaneously in 16 cycles of C-FISH. Quasar 570 labeled decoding probes were used in the odd hybridization cycles to stain GAPDH and in the even cycle to stain Ki67; Cy5 labeled decoding probes were used in the odd hybridization cycles to stain Ki67 and in the even cycle to stain GAPDH. Scale bars, 5 µm.

**Figure 7:**
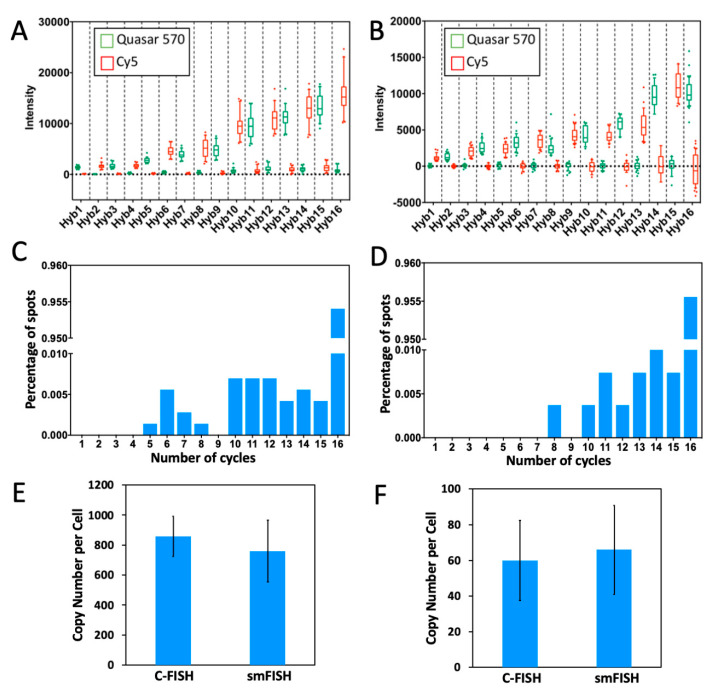
(**A**) Intensity distribution of the GAPDH transcripts (*n* = 45 spots) in 16 cycles of C-FISH. In each cycle, the signal intensities in Quasar 570 and Cy5 channels are shown in green and red, respectively. (**B**) Intensity distribution of the Ki67 transcripts (*n* = 45 spots) in 16 cycles of C-FISH. In each cycle, the signal intensities in Quasar 570 and Cy5 channels are shown in green and red, respectively. (**C**) Fraction of GAPDH spots (*n* = 1000 spots) appearing in different number of C-FISH cycles. (**D**) Fraction of Ki67 spots (*n* = 1000 spots) appearing in different number of C-FISH cycles. (**E**) Comparison of the GAPDH mean copy number per cell (*n* = 30 cells) of transcripts measured by C-FISH and conventional single molecule FISH (smFISH) (*p* > 0.41). (**F**) Comparison of the Ki67 mean copy number per cell (*n* = 30 cells) of transcripts measured by C-FISH and conventional single molecule FISH (smFISH) (*p* > 0.55).

To demonstrate the feasibility of applying C-FISH for multiplexed RNA analysis, we stained mRNA GAPDH and Ki67 (Figure 6) simultaneously in 16 consecutive hybridization cycles. In the odd cycles, GAPDH and Ki67 transcripts were detected with Quasar 570 and Cy5 labeled probes, respectively. And in the even cycles, mRNA GAPDH and Ki67 were stained with Cy5 and Quasar 570, respectively. Throughout the 16 cycles, all the analyzed FISH spots were only observed in the correct fluorescence channel as designed, suggesting the high-specificity and minimum cross-hybridization of the decoding probes for different targets. We also quantified the signal intensities of the mRNA spots in both fluorescence channels in each cycle (Figure 7A,B). The signal from the correct fluorescence channel is increased with the cycle number, while the signal intensities from the other channel remains at the background level. This enhanced signal to background ratio facilitates the spot identification in the latter cycles. As a result, almost all of spots colocalized in the first two cycles reoccur in the third cycle, and more than 95% of these spots are successfully detected in all the following cycles (Figure 7C,D). We use the number of spots appearing in all the 16 cycles to calculate the transcript copy number per cell. The generated results are consistent with the ones produced by conventional smFISH (Figure 7E,F) and the ones reported using RNA-Seq [27]. These data suggest that C-FISH enables multiplexed RNA analysis in single cells in situ, with over 95% detection sensitivity and close to “0” raw data error rate. 

**Figure 8:**
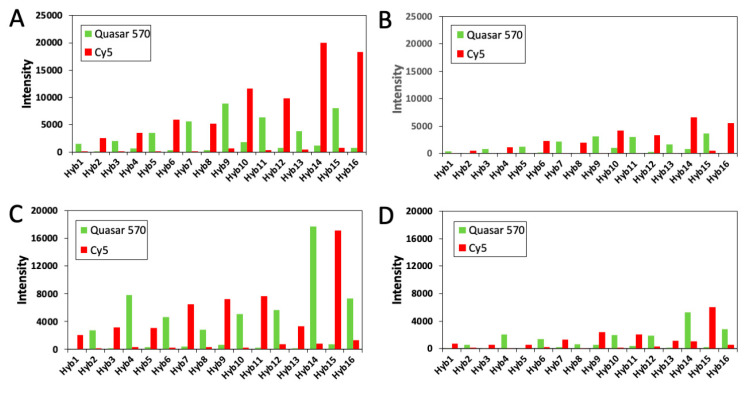
(**A**) Signal intensity profiles of a GAPDH transcript with strong intensities in Figure 6. (**B**) Signal intensity profiles of a GAPDH transcript with weak intensities in Figure 6. (**C**) Signal intensity profiles of a Ki67 transcript with strong intensities in Figure 6. (**D**) Signal intensity profiles of a Ki67 transcript with weak intensities in Figure 6. The signal intensities in the Quasar 570 and Cy5 channels are shown in green and red, respectively.

In every cycle of SeqFISH and MER-FISH, only a fraction of transcripts are stained, while other mRNA targets are not labeled. To determine which FISH spots are actually the newly stained spots in a specific cycle, an artificial threshold must be made. Nonetheless, because of the imperfect hybridization efficiency, proteins bound to the RNA targets, RNA secondary structures, and other factors, the staining intensities from the same RNA species can be different. Consequently, the artificial threshold can lead to false positive and false negative signals, if the signal leftover from the previous cycles are too strong or the real staining signal is too weak. When these errors are accumulated in each cycle, the overall error rate to generate the whole color sequence can be quite significant. In contrast, all the nucleic acid targets are stained in each cycle of C-FISH. Instead of applying a threshold, we compare the intensities of the FISH spots in all the fluorescence channels to determine the identities of the decoding probes. In this way, the true color sequences of both strong spots (Figure 8A,C) and weak spots (Figure 8B,D) can be accurately deciphered. These results demonstrate that C-FISH eliminates the errors generated by the artificial threshold, and improves the analysis accuracy and detection sensitivity. 

## 3. Discussion

We designed and prepared the C-FISH probes, and applied them for multiplexed nucleic acids analysis in individual cells in their natural spatial context. In comparison with the current technologies, our approach has several unique advantages. (i) As tens of thousands of different color sequences can be generated within a small number of analysis cycles, C-FISH has the potential to enable genome- and transcriptome-wide analysis. (ii) Rather than reverse transcribe the RNA into cDNA, C-FISH hybridizes the probes to the mRNA directly. As a result, our approach has single-molecule sensitivity. (iii) With multiple decoding probes hybridized to every single probe applied in the previous cycle, the staining signals are amplified as the cycle number increases. As a result, C-FISH solves the problem of signal loss in the latter cycles. Additionally, as the averaged signal intensities per cycle are amplified by 5 to 6 times (Figure 7), the imaging speed of C-FISH can also be enhanced by 5 to 6 fold, compared with SeqFISH or MERFISH. (iv) Removing the requirement of using artificial threshold to identify the FISH spots, C-FISH allows all the strong and weak spots to be profiled accurately, which significantly lowers the error rates and improves the detection sensitivity.

The multiplexing capacity of C-FISH depends on two factors: the number of analysis cycles and the number of fluorophores applied in each cycle. As we have shown, at least 16 or 9 cycles of C-FISH can be carried out with high analysis accuracy to quantify RNA and DNA, respectively. With four fluorophores used in each hybridization cycle, just eight cycles of C-FISH (4^8^ = 65,536) will enable genome- or transcriptome-wide analysis. Furthermore, to allow more fluorophores to be applied in one cycle, multispectral fluorophore-conjugated [28,29,30] decoding probes can be used and distinguished by hyperspectral imaging [31]. As a result, the cycle number and assay time can be further reduced.

The C-FISH approach reported here promises to advance our understanding of many biological systems. For example, the spatial transcriptomics and genomics analysis of tumor tissues will allow us to study the mechanisms of cancer initiation, progression and metastasis. Profiling individual cells in brain tissues will provide new insights into our understating of brain function and diseases. Additionally, by comparing the immune cells catalogue under the different conditions, we can investigate how the immune systems respond and evolve after antigen activation. In addition, C-FISH can be applied to monitor embryo development at various stages, to investigate the molecular mechanisms of stem cell differentiation and organ formation. Furthermore, by visualizing tens of thousands of varied genomic loci in single cells, we can study how cells establish and regulate the 3-D architecture of the genome, and how 3-D genome architecture regulates gene expression. 

One potential limitation of C-FISH is that the large complex formed by decoding probes will interfere with the precise analysis of nucleic acids in high density. To address this issue, super-resolution microscopy [32] or expansion microscopy [33] can be applied to differentiate those optically crowded spots. Additionally, the size of the probe complex can also be reduced by using the decoding probes with just one binding site in the later cycles, after the desired signal to background ratio has been achieved. Another potential issue of C-FISH is that it requires a large number of orthogonal decoding probes for comprehensive nucleic acids profiling. In total, 240,000 orthogonal sequences [34] have been identified, validated and shown minimum cross-hybridization. The decoding probes designed from those orthogonal sequences will allow 30,000 nucleic acids to be profiled in 8 C-FISH cycles. In SeqFISH or MERFISH, each target is hybridized by 30–40 pre-decoding probes; while C-FISH requires ~8 additional decoding probes per target. Therefore, the total number of probes used in C-FISH is comparable to that in SeqFISH or MERFISH. And all the probes can be prepared cost-effectively on a microarray slide by massively parallel synthesis [35]. Furthermore, our group recently demonstrated that the decoding probes can be efficiently removed by strand displacement reactions [15]. With this approach, we can stain the targets with the same decoding probes in the later cycles, once the preferred signal intensities are already achieved. In this way, the number of required decoding probes, as well as the size of the probe complex, can be significantly reduced. With these further developments, C-FISH has the potential to enable single-cell in situ genomic or transcriptomic analysis. Finally, C-FISH can be combined with the single cell protein analysis technologies [36,37] to allow integrated DNA, RNA and protein analysis in individual cells in situ. This comprehensive molecular imaging platform will have wide applications in systems biology and precision medicine. 

## 4. Materials and Methods 

### 4.1. General Information

Chemicals, solvents and bioreagents were purchased from MilliporeSigma (Burlington, MA, USA) or Thermo Fisher Scientific (Waltham, MA, USA), unless otherwise indicated. All the reagents were used without further purification, unless otherwise noted. All solutions were prepared as RNase-free.

### 4.2. Cell Culture

HeLa CCL-2 cells (ATCC) were maintained in a humidified incubator at 37 °C with 5% CO_2_, and were supplied with DMEM (Dulbecco’s Modified Eagle’s Medium) supplemented with 10% fetal bovine serum, 10 U/mL penicillin and 100 g/mL. Cells were subcultured on 8-well chambered coverglass to reach 60% confluency until experiments were performed.

### 4.3. Cell Fixation

After washing with 1X PBS at room temperature for 5 min, the cultured HeLa cells were fixed with 4% formaldehyde (Polusciences) in 1X PBS at room temperature for 10 min. Then, the cells were washed twice with 1X PBS at room temperature, each for 5 min. Subsequently, the cells were permeabilized overnight with 70% (*v/v*) ethanol at 4 °C.

### 4.4. Probe Design

All the RNA probes and the DNA decoding probes are 70 nt long containing three 20 nt sequences: (i) the first 20 nt sequence to hybridize to the target or the probe used in the previous cycle, and (ii) the second and third 20 nt shared sequences to recruit the probe in the following cycle. Two 5T spacers were inserted between these three 20 nt sequences to separate them from each other. The 45 nt DNA pre-decoding probes are composed of a 20 nt target-binding sequence and a 20 nt pre-decoding probe-binding sequence, separated by a flanking 5T spacer. The target-binding sequences for GAPDH and Ki67 transcripts were designed by the Stellaris Probe Designer (Biosearch Technology). The target-binding sequences for genomic locus 4p16.1 were designed as described in [38]. The sequences of all the probes are provided in Supplementary Material Table S1.

### 4.5. Probe Preparation

As each nucleic acid target is hybridized by over 30 pre-decoding probes simultaneously to provide real signals, the small percentage of the impure probes generated during oligonucleotide synthesis will not result in detectable false positive signals. Therefore, the pre-decoding probes are used directly, without further purification. Pre-decoding oligonucleotides (IDT) for the same nucleic acid target were combined and stored at 4 °C in the pre-decoding probe stock solution (10 mM in 1% 1X Tris EDTA, pH 8.0). 

To the solution of 1 nmol of decoding probe in 3 µL of water was added 3 µL of 1 M NaHCO_3_ aqueous solution and 5 µL of 20 mM Alexa 488, Quasar 570 (Biosearch) or Cy5 (AAT Bioquest) N-hydroxysuccinimide (NHS) ester in DMF. The reaction mixture was incubated for 2 h at room temperature. Subsequently, the oligonucleotides were purified with a nucleotide removal kit (Qiagen). The fluorescently labeled oligonucleotides were further purified using HPLC equipped with a C18 column (Aligent). With a dual wavelength detector, DNA absorption was detected at 260 nm and the fluorophore absorption was detected at 496 nm for Alexa 488, 548 nm for Quasar 570 and 650 nm for Cy5. Triethyl ammonium acetate (0.1 M, pH 6.5) and acetonitrile (pH 6.5) were used as Buffer A and B, respectively. The HPLC gradient ranged from 7% to 30% of Buffer B in 30 min, then at 70% of Buffer B for 10 min, finally at 7% of Buffer B for 10 min. The flow rate was at 1 mL/min. After drying the collected fraction with a Savant SpeedVac Concentrator, the labeled decoding probes were stored in 100 µL of decoding probe stock solution (1% 1X Tris EDTA, pH 8.0) at 4 °C. Through chemical conjugation and HPLC purification, the small percentage of the impure decoding probes generated during oligonucleotide synthesis are removed, and will not interfere with the designed hybridization reactions.

### 4.6. RNA Pre-Decoding Hybridization

To prepare the RNA pre-decoding hybridization solution, 1 µL of the pre-decoding probe stock solution was added to 100 µL of pre-decoding hybridization buffer (10% formamide, 1 mg/mL Escherichia coli tRNA, 100 mg/mL dextran sulfate, 20 µg/mL bovine serum albumin and 2 mM vanadyl ribonucleoside complex in 2X saline-sodium citrate (SSC)). 

After incubation with wash buffer (10% formamide and 2 mM vanadyl ribonucleoside complex in 2X SSC) at room temperature for 5 min, the fixed and permeabilized HeLa cells were incubated with the RNA pre-decoding hybridization solution overnight at 37 °C. Subsequently, the cells were washed three times with wash buffer at 37 °C each for 30 min.

### 4.7. DNA Pre-Decoding Hybridization

To prepare the DNA pre-decoding hybridization solution, 1 µL of the pre-decoding probe stock solution was added to 100 µL of DNA pre-decoding hybridization buffer (50% formamide and 100 mg/mL dextran sulfate in 2X SSCT (0.1% Tween-20 in 2X SSC)). 

The fixed HeLa cells were first washed once with 1X PBS for 1 min, then incubated with 1X PBST (0.1% Tween-20 in 1X PBS) for 1 min. The cells were then incubated with 1X PBS + 0.5%(*v/v*) Triton-X100 for 10 min, followed by 1X PBST wash for 2 min. Subsequently, the cells were treated with 0.4 mg/mL RNase A in 1X PBST for 15 min at 37 °C and washed with 1X PBS for 5 min. The cells were then incubated with 0.1 M HCl for 5 min, and washed with 2x SSCT three times for 2 min. The cells were then washed with 70% formamide in 2X SSCT for 5 min, and incubated in the same solution for 20 min at 78 °C, followed by same solution for 20 min at 60 °C. The cells were then cooled to room temperature. The cells were subsequently incubated with 70% formamide in 2X SSCT for 15 min at 78 °C. Afterwards, the cells were incubated with pre-decoding hybridization solution for 10 min at 78 °C. Then, the cells were incubated in a humidified chamber at 37 °C overnight. The cells were washed with 2X SSC for 15 min at 60 °C, then 10 min at room temperature, then with 0.2X SSC for 10 min at room temperature. 

### 4.8. Consecutive RNA and DNA FISH

To prepare the RNA and DNA decoding hybridization solution, 5 µL of the decoding probe stock solution was added to 100 µL of decoding hybridization buffer (10% formamide, 2 mM vanadyl ribonucleoside complex and 100 mg/mL dextran sulfate in 2X SSC). 

Following pre-decoding hybridization, the HeLa cells were incubated with the decoding hybridization solution for 30 min at 37 °C. Then, the cells were washed with wash buffer for 30 min at 37 °C and incubated with 4′,6-diamidino-2-phenylindole (DAPI) (5 ng/mL in wash buffer) at 37 °C for 30 min to stain the nucleus. The stained cells were washed with GLOX buffer (10 mM Tris HCl and 0.4% glucose in 2X SSC) at room temperature for 2 min, and then imaged in GLOX solution (1% catalase and 0.37 mg/mL glucose oxidase in GLOX buffer). Following image capture, Alexa 488, Quasar 570 or Cy5 was photobleached with their corresponding filters at each z step for 60 s, 20 s and 5 s, respectively. During photobleaching, the cells were incubated in photobleaching buffer (2 mM vanadyl ribonucleoside complex in 2X SSC). To remove the free radicals, photobleaching buffer was changed every 3 min.

### 4.9. Consecutive FISH Imaging

All the images were captured using NIS-Elements Imaging software and a Nikon Ti-E epifluorescence microscope, which is equipped with a 100X objective and a CoolSNAP HQ2 camera. The stained cells were imaged with a 0.3 µm z spacing and 5 µm range. Alexa 488, Quasar 570 and Cy5 were imaged using Chroma filters 49011, 49004 and 49009, respectively. 

### 4.10. Image Analysis

Based on one unique fluorescent spot, which appears in every cycle, all the images collected from different hybridization cycles were aligned to the first cycle coordination system. The locations of the fluorescent spots detected in each cycle were then determined by SpotDetector [39]. The spots identified in different cycles with a distance of less than 320 nm (2 pixels) were defined as colocalized spots. The *p*-values (student’s *t* test) were calculated using Excel (Microsoft).

## Supplementary Materials

The Supplementary Materials are available online. Table S1: Probe sequences.
